# Ultrasonic shear wave elastography predicts the quality of the residual tendon before the rotator cuff repair

**DOI:** 10.1186/s13244-024-01642-7

**Published:** 2024-03-14

**Authors:** Xianghui Chen, Siming Chen, Fei Zhang, Yaqiong Zhu, Dan Yi, Hong Xu, Jie Tang, Qiang Zhang, Yuexiang Wang

**Affiliations:** 1 Department of Ultrasound, Third Medical Center of PLA General Hospital, Yongding Road 69, Beijing, 100853 China; 2 Department of Ultrasound, The First Medical Center of PLA General Hospital, Fuxing Road 28, Beijing, 100853 China; 3 Department of Orthopedics, The First Medical Center of PLA General Hospital, Fuxing Road 28, Beijing, 100853 China

**Keywords:** Arthroscopy, Rotator cuff injuries, Shear wave elastography, Shear wave velocity, Ultrasonography

## Abstract

**Background and purpose:**

Effective evaluation of rotator cuff tear residual tendon quality is the key to surgical repair. However, until now, the evaluation of rotator cuff tissue by ultrasonic shear wave elasticity (SWE) has been controversial. This prospective study analyzed the association between preoperative SWE and arthroscopic residual tendon quality scores.

**Methods:**

The shear wave velocity (SWV) of the deltoid muscle, the supraspinatus tendon, and the supraspinatus muscle were measured in full-thickness rotator cuff tear patients. Tendon quality was scored according to tear size, tendon margin, tendon thickness, and footprint coverage during arthroscopy. The arthroscopic scores were used as the gold standard, and the SWV ratio of tendon and muscle (supraspinatus tendon/deltoid and supraspinatus muscle/deltoid) were calculated and correlated with the arthroscopic scores.

**Result:**

Eighty-nine patients (129 shoulders) were enrolled, including 89 operation shoulders and 40 control shoulders. In the group of operation shoulders, both the SWV ratios of tendon (SWV-RT) and the SWV ratio of muscle (SWV-RM) were negatively correlated with arthroscopic scores (The correlation coefficient (R) ranged from -0.722 to -0.884 and -0.569 to -0.689). The SWV-RT and SWV-RM of the operation shoulders were significantly lower than that of the control shoulders (*p* < 0.05).

**Conclusion:**

SWE could be used to predict the quality of the residual tendon before the rotator cuff repair. SWV of the supraspinatus tendon and muscle was a useful parameter to predict the quality of the residual tendon.

**Critical relevance statement:**

Measuring the shear wave velocity of the supraspinatus tendon and muscle with SWE is useful for predicting the quality of the residual tendon which is one of the key factors for a successful rotator cuff repair.

**Key points:**

• Evaluating the quality of the residual tendon is important before surgery.

• Elasticity measurements were negatively correlated with the arthroscopic score.

• SWE is useful for predicting the quality of the residual tendon.

**Graphical Abstract:**

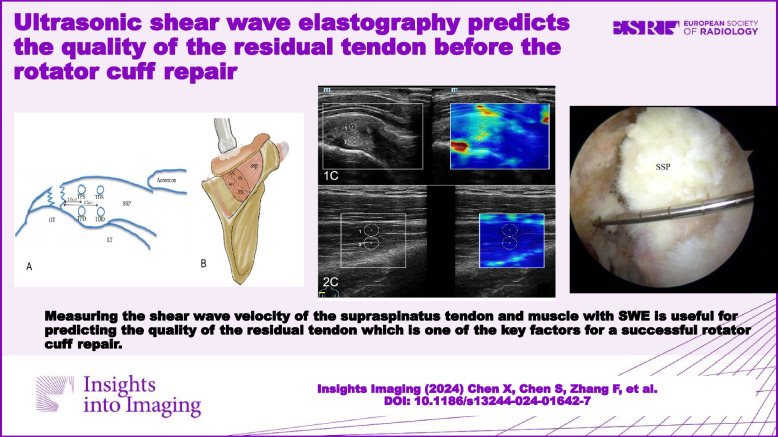

## Introduction

Rotator cuff tear (RCT) is a main cause of shoulder pain and dysfunction with an incidence of more than 20% [1, 2] and can seriously affects patients’ daily life quality [3]. Surgical treatment is the main treatment for full-thickness RCT [4].

RCT repair is primarily performed to restore the normal anatomy and tissue mechanics of the rotator cuff by completely repairing the residual tendon to the footprint [5]. However, not all RCTs can achieve complete repair [5, 6]. Poor muscle or tendon extensibility and large tensile stresses at the repair site might lead to increased postoperative pain and risk of re-tear for the patients [7, 8]. Therefore, noninvasively evaluating the quality of the residual tendon and its muscle before the RCT repair has become the most concerned issue for surgeons.

Preoperative imaging methods for RCTs mainly included MRI and conventional ultrasound, but neither of these methodologies provide information on tissue mechanics [6]. Surgeons can only assess the repairability of RCTs by relying on indirect imaging signs, such as tear size, tendon retraction, level of muscle fat infiltration, and degree of muscle atrophy [9].

Ultrasonic shear wave elastography (SWE) can provide mechanical tissue information and has been applied to the study of thyroid, breast, liver, prostate, lymph nodes, and other related diseases [10, 11]. SWE has the potential to quantify residual tendon quality before surgery to help surgeons understand tissue mechanics and the difficulty of repair. However, SWE in assessing the quality of RCT remnant tendon is still controversial. Some studies [11–14] have suggested that SWE was affected by many factors, such as equipment and examination conditions, depth of measurement, and patient positioning. One study [15] showed that the ultrasonic elasticity measurement was not significantly correlated with the rotator cuff tear size, tendon retraction, and fat infiltration. However, another retrospective study [16] has shown that SWE could provide mechanical tissue information that conventional two-dimensional ultrasound could not provide. The study of Krepkin et al. [17] showed that the shear wave velocity (SWV) of tendon was negatively correlated with the size of the tear (*R* = -0.79), which suggested that the SWV measured by SWE could be used as a quantitative index to evaluate the quality of the residual tendon.

The purpose of this study was to measure the rotator cuff tissue stiffness prior to the arthroscopy by a standardized SWE procedure and correlate these results with a semi-quantitative arthroscopic score of the residual tendon quality. We hypothesized that rotator cuff tissue stiffness measured by SWE would correlate with the quality of residual tendon and thus could be used to predict the repairability of RCT.

## Materials and methods

Our prospective study was approved by the Ethics Committee of our hospital and the Chinese Clinical Trial Register. Informed consent was obtained from all participants.

### Patients

Ninety-five patients who were diagnosed with full-thickness RCT by MRI and/or conventional ultrasound in our institution between March 2022 and January 2023 and prepared for arthroscopy were prospectively enrolled. After excluding 1 patient with shoulder joint operation history, 1 patient with shoulder dislocation, and 4 patients with calcifying tendinopathy, 89 patients were finally included.

**Fig. 1:**
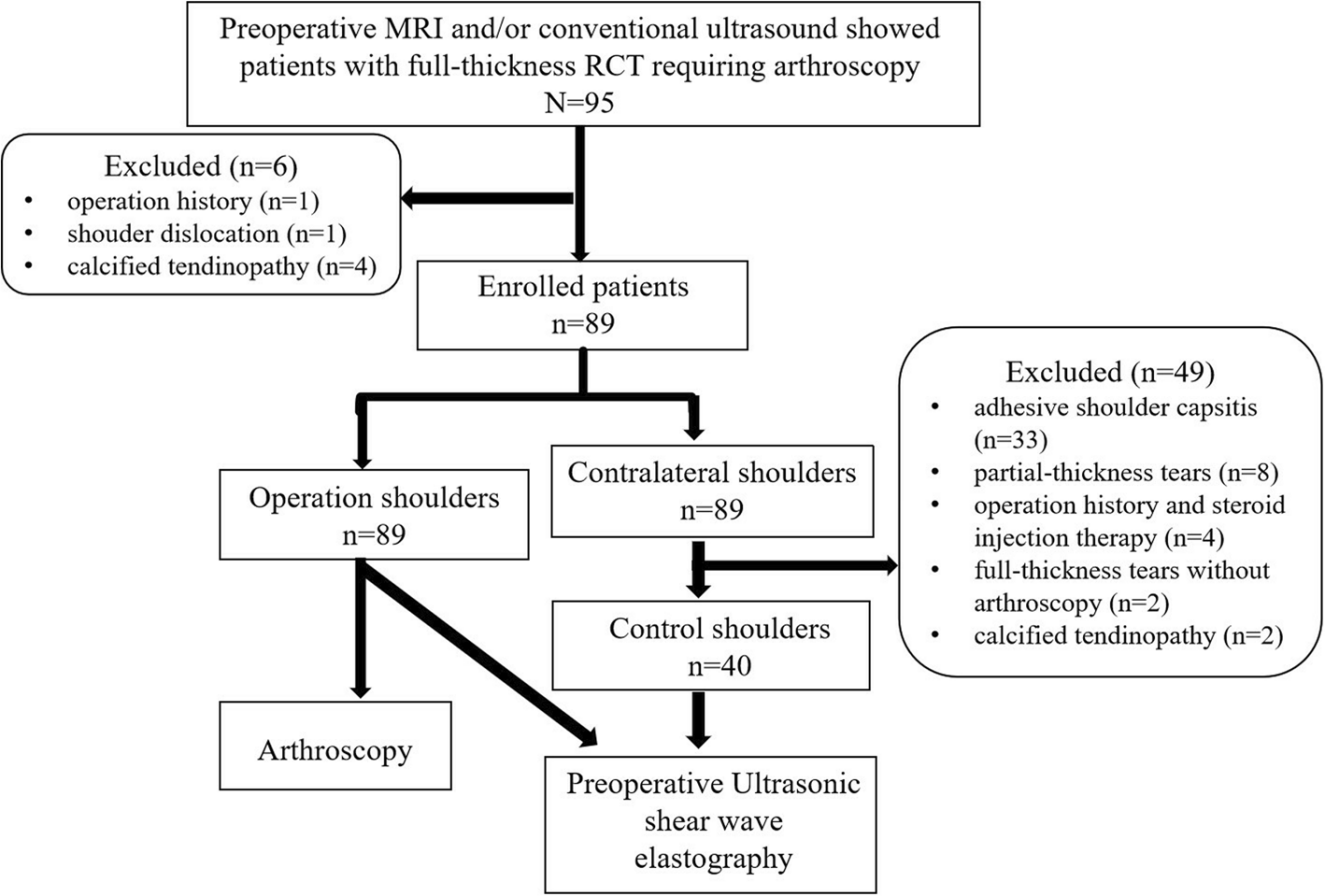
Flow diagram of study population. RCT, rotator cuff tear

The included patients were divided into 89 operation shoulders and 89 contralateral shoulders. The operative shoulders had shoulder pain and/or shoulder joint dysfunction, and full-thickness RCT was confirmed by MRI or ultrasound examination which required arthroscopic repair. In the contralateral shoulders, 33 shoulders were excluded for adhesive shoulder capsulitis, 8 for partial-thickness tears, 4 for operation history and steroid injection therapy, 2 for full-thickness tears without arthroscopy repair, and 2 for calcifying tendinopathy; thus, the remaining 40 normal shoulders with no pain symptoms and shoulder joint dysfunction, in which ultrasound examination showed normal rotator cuff, were used as the control shoulders (Fig. 1).

### Consistency assessment of SWE

Ten healthy subjects with normal bilateral shoulder joints were selected, with an average age of 29.70 ± 4.59 years, including 4 males and 6 females. The first bilateral shoulder SWE measurement was performed by one radiologist with 6 years of experience and another radiologist with 9 years of experience. The subjects were seated with relaxed shoulders and natural flexion of the ipsilateral upper limb. The palm was placed upward on the ipsilateral thigh. SWV was measured 1 cm from the attachment of the supraspinatus tendon of the shoulder, and the two radiologists were unaware of each other’s measurement results. One week later, a second measurement was taken by the radiologists in these ten subjects with the same SWV examination method. Intra- and inter-observer consistency of SWV measurements was assessed.

### Ultrasound SWE examination

One radiologist with six years of experience in ultrasound diagnosis performed all ultrasound examinations, using ultrasound systems (Mindray: Resona R9, Shenzhen, China) with a L11-3U liner transducer (3–11 MHz). The radiologist knew that each patient had an RCT by performing a conventional ultrasound examination and checked if the patient’s opposite shoulder was normal. Then, SWE was performed on the affected shoulder and the opposite normal shoulder.

Any high-intensity shoulder movement was avoided for at least half an hour before the ultrasonic assessment. According to the shoulder ultrasound guidelines [18], the rotator cuff structure was examined sequentially. The patient was seated in two positions (natural and modified crass). The natural position required the patient’s shoulder in a relaxed state with 0° abduction and neutral rotation and put the palm upward on the ipsilateral thigh. The modified crass position required the patient’s spine to be naturally upright, with the palm placed on the ipsilateral hip, the elbow backward, and the shoulder joint rotated externally.

**Fig. 2:**
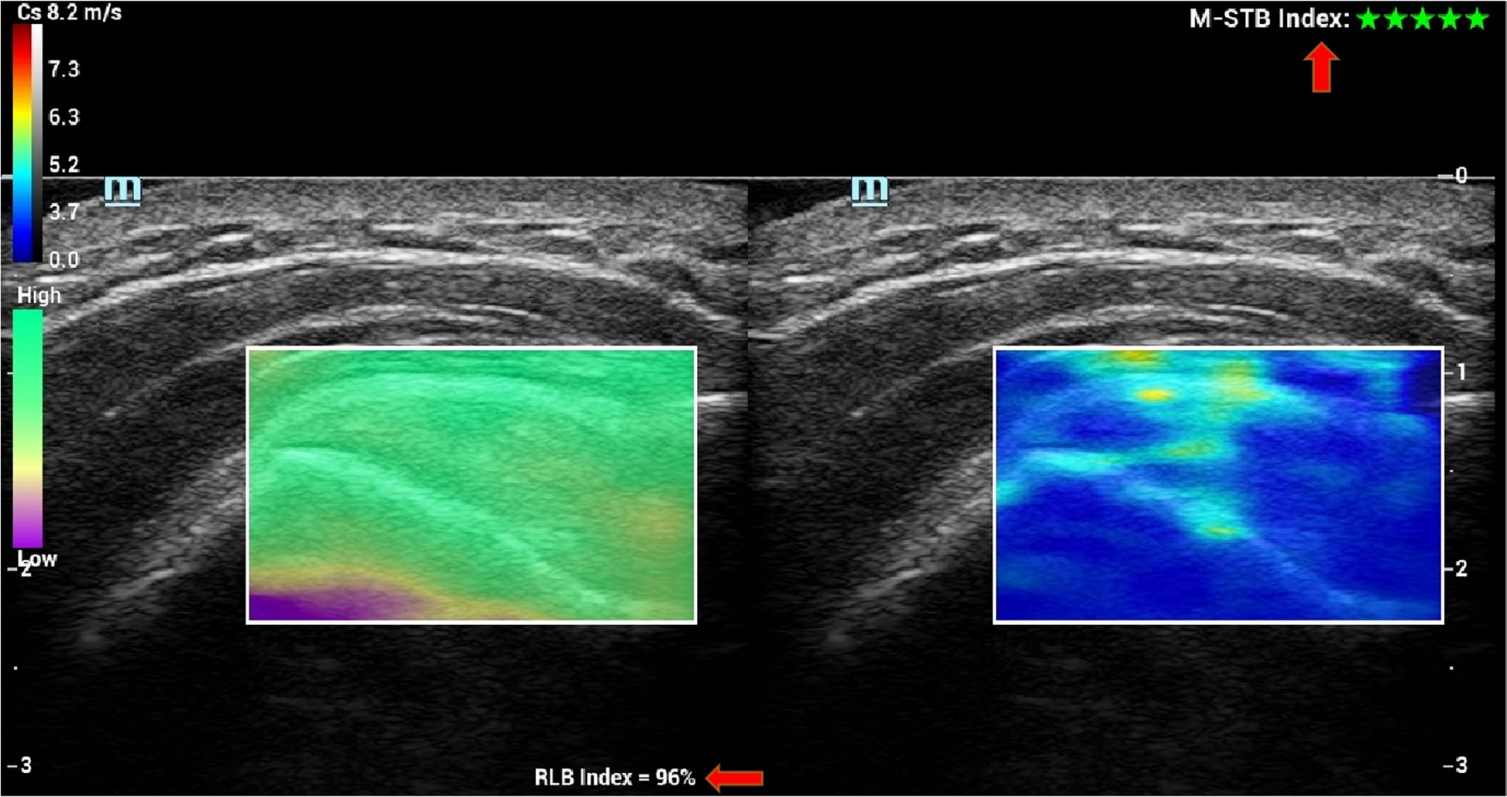
Image quality control diagram of SWE measurement. SWV of normal supraspinatus shoulder tendon was measured with an image M-Stability index of five stars (arrow) and an image reliability index of 96% (arrow). In the upper color scale, the SWV measurement range was set to 0.0–8.2 m/s, and red indicated the maximum stiffness, while blue indicated the minimum stiffness. On the lower color scale, green indicated the most reliable image, and purple indicated the least reliable image

**Fig. 3:**
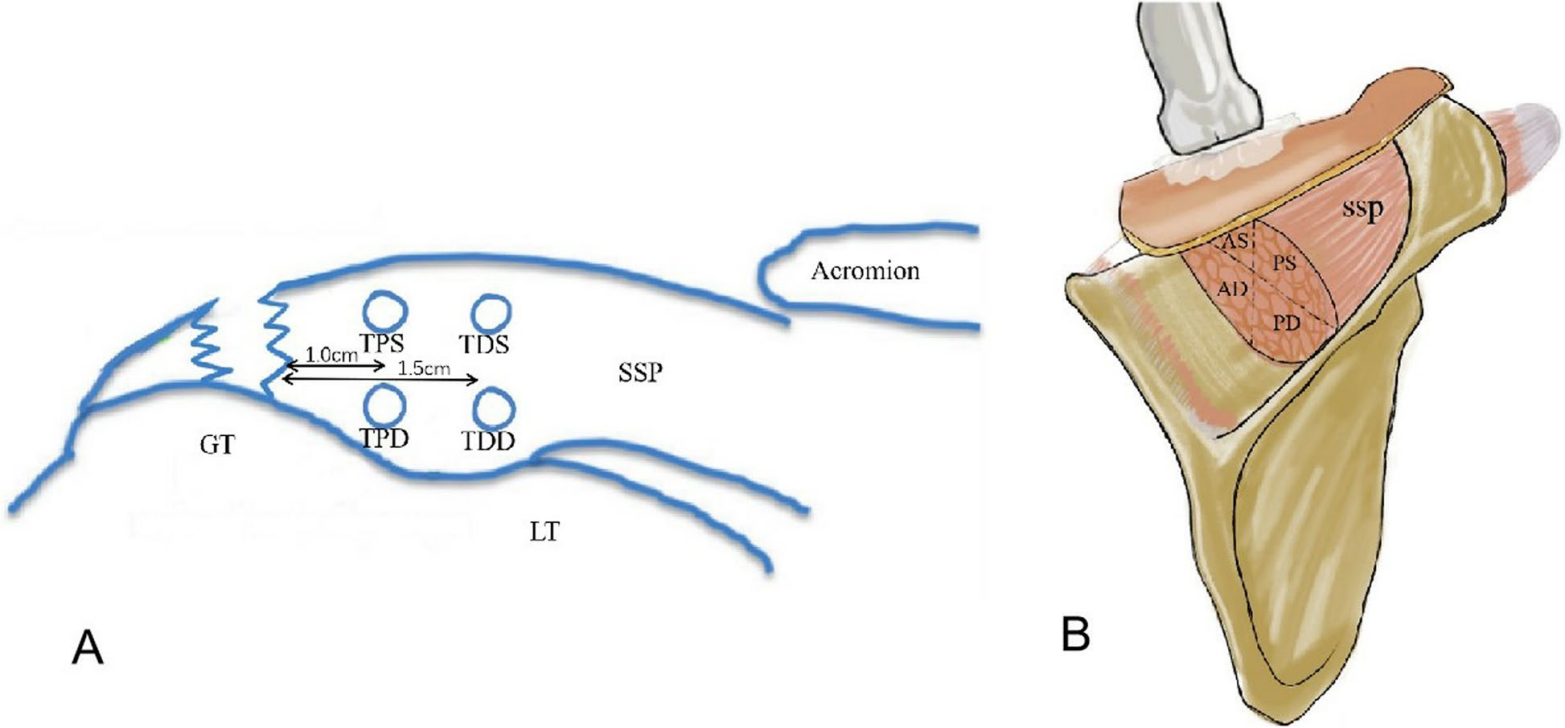
Diagram of the site of the supraspinatus tendon (**A**) and muscle (**B**) where the SWE was measured. *SSP *supraspinatus; *GT *greater tubercle of humerus; *LT *lesser tubercles of humerus; *TPS *proximal-superficial of tendon; *TPD *proximal-deep of tendon; *TDS *distal-superficial of tendon; *TDD *distal-deep of tendon; *AS *anterior-superficial of muscle; *AD *anterior-deep of muscle; *PS *posterior-superficial of muscle; *PD *posterior-deep of muscle

**Fig. 4:**
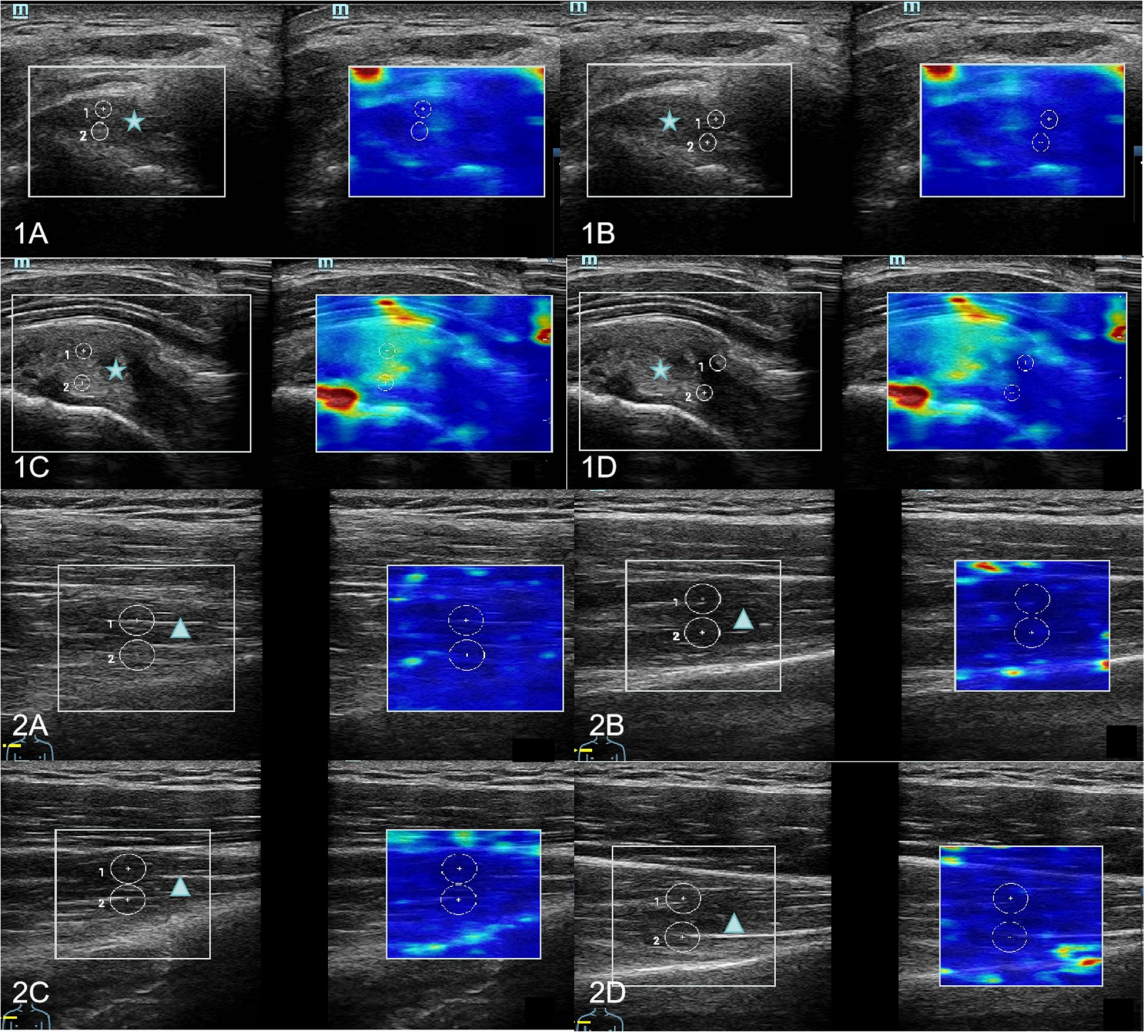
Ultrasound image of the supraspinatus tendon and muscle measured by SWE. **A**, **B** Natural position SWE measured SWV at four sites of the supraspinatus tendon; 1C-1D: modified crass position SWE measured SWV at four sites of the supraspinatus tendon. **A**, **B** Natural position SWE measured SWV at four sites of the supraspinatus muscle; 2C-2D: modified crass position SWE measured SWV at four sites of the supraspinatus muscle. Pentagram: residual tendon after tear of supraspinatus tendon. Triangle: supraspinatus muscle

The ultrasound gain range was set to 80–90. After the conventional ultrasound scan, SWE was performed on the affected shoulder and on the opposite normal shoulder respectively under two positional conditions. The focus of ultrasound was located at the site of SWE measurement. The image depth was 3 cm for the supraspinatus tendon and 5 cm for the supraspinatus muscle. The SWV measurement range was set to 0.0–8.2 m/s. The SWE measurement site was placed in the middle of the region of interest (ROI), and the ROI was set as 1–2 mm sample volumes for supraspinatus tendons and 5 mm sample volumes for supraspinatus muscles. The image was frozen when the image M-Stability index of the SWE examination suggested five stars, and the image reliability index was more than 95% (Fig. 2). The transducer was aligned with the tendon fibers, ultrasound gel was used to obtain contact between the skin surface and the transducer, and care was taken to make the minimum and even contact pressure to achieve good image quality. The SWE mode was activated to measure the SWV of the middle tract of the deltoid muscle, proximal-superficial (TPS) and proximal-deep (TPD) site of the supraspinatus tendon at 1.0 cm proximal to the torn edge, and distal-superficial (TDS) and distal-deep (TDD) of the supraspinatus tendon at 1.5 cm proximal to the torn edge, respectively (Figs. 3a and 4). Then, the SWV of the supraspinatus muscle was measured at 4 sites with SWE: anterior-superficial (AS), anterior-deep (AD), posterior-superficial (PS), and posterior-deep (PD) (Figs. 3b and 4). The SWV ratio (SWV-RT) of tendon (supraspinatus tendon/deltoid muscle) and the SWV ratio (SWV-RM) of muscle (supraspinatus muscle/deltoid muscle) were calculated for statistical analysis. All SWE values were measured 3 times, and the mean value was recorded.

### Arthroscopic evaluation

Shoulder arthroscopy was performed with the patient under general anesthesia in the lateral position. The arthroscopy was performed through a posterior pathway.

**Fig. 5:**
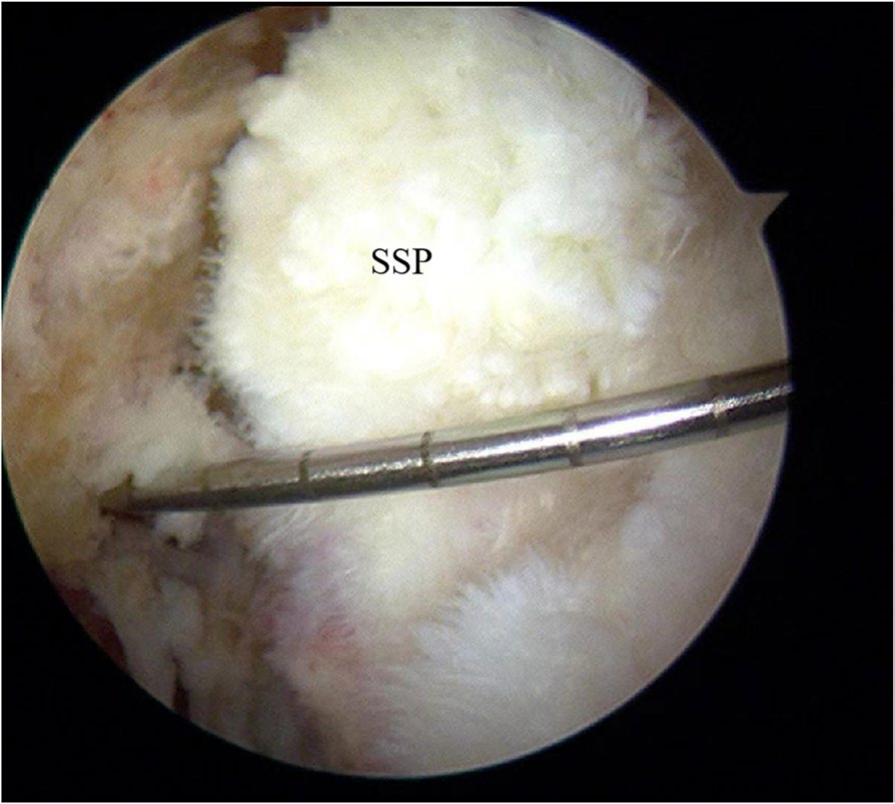
The tear of supraspinatus tendon (SSP) was seen in arthroscopy. A probe was used, and according to the scale on the probe (adjacent scale 0.5 cm), the tear size was measured as 2.5 cm, indicating a medium tear. The margin of the residual tendon was severe fraying, with normal thickness, and could completely cover the footprint area. The arthroscopic score was calculated as 4 points

The quality of the residual tendon was evaluated by a semi-quantitative scoring method, which evaluated the tear size, residual tendon margin, residual tendon thickness, and footprint coverage. As the score increases, the quality of the residual tendon decreases. The tear size was measured according to the method described in previous literature [19]. One probe was used in arthroscopy, and the tear size was measured according to the scale on the probe (adjacent scales were 0.5 cm). The tear size was defined as a small tear (maximum diameter ≤ 1 cm), a medium tear (maximum diameter > 1 cm to ≤ 3 cm), or a large tear (maximum diameter > 3 cm), which was scored as 1, 2, and 3 points respectively. Regarding the edge of the residual tendon, 0 points were scored for sharp and fresh edges of the residual tendon, 1 point for some fraying, and 2 points for severe fraying. Regarding the thickness of the residual tendon, 0 points were scored for the residual tendon with normal thickness, 1 point was for the tendon thinner less than 1/2 normal tendon thickness, and 2 points was for the tendon thinner more than 1/2 normal tendon thickness. Regarding the footprint coverage, 0 points were scored for the residual tendon that could completely cover the footprint area. On the contrary, partial coverage and failure to cover the footprint area which indicated poor tendon quality were scored 2 and 3 points respectively (Fig. 5 and Table 1).

**Table 1:** Quality evaluation of residual tendon during the shoulder arthroscopy

** Item (Total 10 points)**	**Score**
**Tear size (3 points)**	
small tear (maximum diameter ≤ 1 cm)	1
medium tear (maximum diameter >1 cm to ≤ 3 cm)	2
large tear (maximum diameter > 3 cm)	3
**Tendon margin** (**2 points**)	
sharp fresh edge	0
some fraying	1
severe fraying	2
**Tendon thickness (2 points)**	
Normal thickness	0
thinner less than 1/2 tendon	1
thinner more than 1/2 tendon	2
**Footprint coverage (3 points)**	
footprint was completely covered	0
half of the footprint was covered	2
footprints couldn’t be covered	3

All the arthroscope evaluations and RCT repairs were performed by a surgeon with 9 years of clinical experience who was unaware of the results of the SWE.

### Statistical analysis

The data were analyzed by IBM SPSS Statistics, version 26.0. The Kappa (*κ*) test was used to analyze the intra and interobserver consistency of SWV measurements. A *κ* value ranging from 0.80 to 1.00 indicated almost perfect agreement and *κ* value = 1.00 indicated perfect agreement. The *κ* value between 0.61 and 0.80 was considered to represent substantial agreement, with values between 0.41 and 0.60 representing moderate agreement and with values between 0.21 and 0.40 representing fair agreement, whereas values < 0.20 were deemed to represent slight agreement. The arthroscopic scores were the gold standard. Spearman correlation was used to analyze the correlation between the ratio of tendon/muscle and the arthroscopic scores in the operation group. The datasets with the highest correlation coefficients (*R*) were used for subsequent comparative analysis. Wilcoxon-Mann-Whitney test was used to compare SWV differences between the operation group and the control group. *p* < 0.05 was considered statistically significant.

## Results

In the pre-study SWE consistency assessment, the intra-observer *κ* value was 0.848 and inter-observer *κ* value was 0.697.

A total of 89 patients with 129 shoulders were included. There were 89 operation shoulders and 40 control shoulders. The average age of the operation shoulders was 57.57 ± 10.64 years, including 34 males and 55 females, 32 left shoulders, and 57 right shoulders. The average age of the control shoulders was 54.63 ± 10.74 years, 21 males and 19 females, 25 left shoulders, and 15 right shoulders. The included patient’s body mass index (BMI) was 24.79 ± 3.00. The time of pain was 6.97 ± 5.74 months.

The arthroscopic scores were 1 point for 5 shoulders, 2 points for 6 shoulders, 3 points for 8 shoulders, 4 points for 12 shoulders, 5 points for 26 shoulders, 6 points for 26 shoulders, 7 points for 5 shoulders, 8 points for 1 shoulder, and 9 points and 10 points for 0 shoulders. The residual tendon was repaired to completely cover the footprint in 88 shoulders and partially cover the footprint in 1 shoulder.

**Fig. 6:**
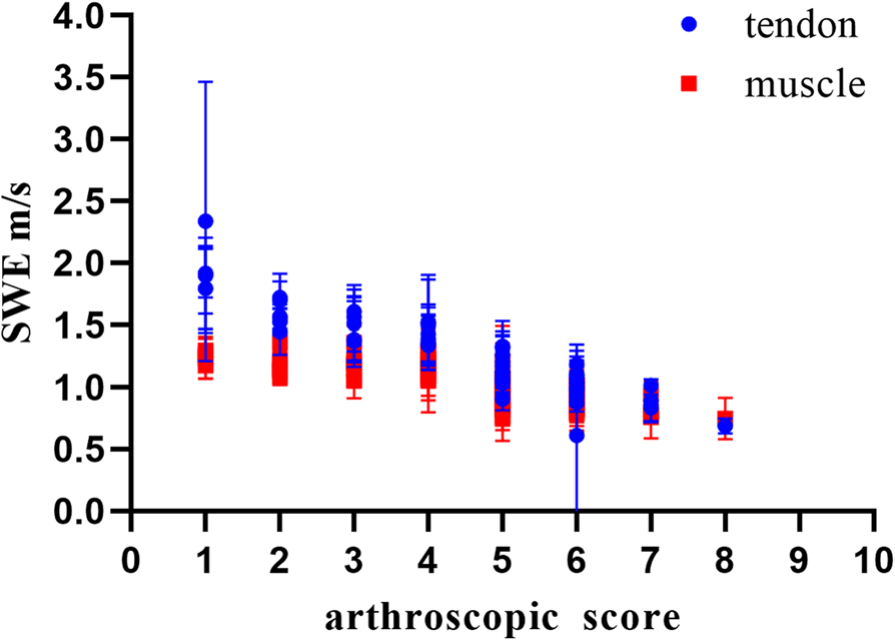
Trend chart of SWE measurements with the arthroscopic scores

The SWV-RT in both natural and modified crass positions were negatively correlated with the arthroscopic scores, with *R* values ranging from -0.722 to -0.884. The SWV-RM in both natural and modified crass positions were negatively correlated with the arthroscopic scores, with *R* values ranging from -0.569 to -0.689. Among all investigated measurements, natural position TDD and modified crass position TPS exhibited the strongest correlations with arthroscopic scores (*R* = -0.884 and -0.852, respectively). Notably, PD of both positions also demonstrated strong correlations with arthroscopic scores (*R* = -0.689 and -0.686) (Fig. 6 and Table 2).

**Table 2:** Correlation between preoperative SWE and arthroscopic scores

	**Correlation** (***R***)	***p* value**
**Full-thickness tears** (***n* = 89**)		
**SWV ratio of tendon**		
Natural position		
TPS	-0.790	< 0.001
TPD	-0.847	< 0.001
TDS	-0.722	< 0.001
TDD	-**0.884**	< 0.001
Modified crass position		
TPS	-**0.852**	< 0.001
TPD	-0.761	< 0.001
TDS	-0.850	< 0.001
TDD	-0.847	< 0.001
**SWV ratio of muscle**		
Natural position		
AS	-0.569	< 0.001
AD	-0.672	< 0.001
PS	-0.616	< 0.001
PD	-**0.689**	< 0.001
Modified crass position		
AS	-0.605	< 0.001
AD	-0.684	< 0.001
PS	-0.590	< 0.001
PD	-**0.686**	< 0.001

*TPS* Proximal-superficial of tendon, *TPD* Proximal deep of tendon, *TDS* Distal-superficial of tendon, *TDD* Distal-deep of tendon, *AS* Anterior-superficial of muscle, *AD* Anterior-deep of muscle, *PS* Posterior-superficial of muscle, *PD* Posterior-deep of muscle

The SWV in the operation shoulders were lower than those in the control shoulders, and the difference between them was statistically significant (*p* < 0.05) (Table 3).

**Table 3:** Comparison of SWE measurements between operation shoulders and control shoulders

**Site**	**Median (IQR)**		**Mean value of rank**		***Z* value**	***p* value**
	**Operation shoulders**	**Control shoulders**	**Operation shoulders**	**Control shoulders**		
**SWV-RT**						
Natural position TDD	1.088 (0.985, 1.279)	1.698 (1.554, 1.899)	47.34	104.30	-8.005	< 0.001
Modified crass position TPS	1.181 (1.067, 1.509)	2.225 (2.076, 2.484)	46.24	106.75	-8.504	< 0.001
**SWV-RM**						
Natural position PD	0.973 (0.802, 1.193)	1.172 (1.115, 1.276)	54.78	87.75	-4.634	< 0.001
Modified crass position PD	1.024 (0.913, 1.124)	1.182 (1.079, 1.254)	53.44	90.71	-5.237	< 0.001

*IQR* Interquartile range, *SWV-RT* SWV ratio of tendon, *SWV-RM* SWV ratio of muscle, *TPS* Proximal-superficial of tendon, *TDD* Distal-deep of tendon, *PD* Posterior-deep of muscle

## Discussion

This study used a semi-quantitative scoring method to assess residual tendon quality during arthroscopy for the first time, and the change of the supraspinatus tendon and muscle stiffness measured by SWE was compared with the arthroscopic scores. Many previous studies [7, 20–22] compared SWE with MRI examination of tear size, tendon retraction degree, muscle fat infiltration, and muscle atrophy to indirectly reflect whether SWE can predict rotator cuff tissue stiffness. However, imaging findings are often helpful for the clinician’s initial diagnosis and preoperative prediction of tendon quality, but the final surgical approach depends on intraoperative findings. In one study [10], the authors used a measuring instrument to record intraoperative traction of the residual tendon being pulled to the insertion and compared the preoperative SWE with this traction. We acknowledge that this was a more efficient approach, but in practice, the surgeon evaluates the quality of the residual tendon during arthroscopy based on tear size, margin, thickness, and whether the residual tendon can fully cover the footprint area under the appropriate tensile stress [23]. Therefore, we used a semi-quantitative scoring method based on intraoperative assessment items to reflect tendon quality, which was also conducive to further clinical promotion.

Due to the irregular shape of the rotator cuff structure, which was heterogeneous and anisotropic compared with homogeneous tissues (such as liver, breast, and thyroid), and the insertion of the supraspinatus tendon at the greater tubercle of the humerus, SWE measurement is susceptible to cortical interferences [12]; therefore, SWV-RT and SWV-RM were used for statistical analysis in our study. Our study showed that the SWV-RT and SWV-RM were negatively correlated with the arthroscopic scores, indicating that the stiffness of rotator cuff tissue gradually decreased with the deterioration of tendon quality. This was similar to the results of previous studies [24–26], suggesting that measurements of elasticity of supraspinatus tendon and supraspinatus muscle can effectively reflect rotator cuff tissue dynamics. As research has progressed, it has been recognized that over-tensioning the residual tendon was detrimental to healing. Intraoperative low-tension repair is a prerequisite for successful rotator cuff repair [27, 28]. SWE has the potential to quantitatively assess the quality of residual tendons before surgery to help surgeons understand the tissue mechanics and the difficulty of repair and plan the operation in advance [29].

In our study, whether in the natural or modified crass position, SWV-RT in four different areas within the residual tendon were strongly correlated with the arthroscopic scores. This finding is consistent with previous findings, suggesting that SWE measurement has the potential to noninvasively assess the changes in tendon elasticity and be useful for the preoperative evaluation of residual tendon quality. Our results also showed that the SWV-RM measured in the PD part of the supraspinatus muscle in both the natural and modified crass positions had the strongest correlation with the arthroscopic scores (-0.689 and -0.686) compared to other parts of the supraspinatus muscle. In the study conducted by Itoigawa et al. [10], SWE measurements were performed in the AS, AD, PS, and PD part of the supraspinatus muscle in 38 patients with full-thickness tear requiring arthroscopic shoulder repair. The study results showed that the SWE of the PD part of the supraspinatus muscle had the strongest correlation with the passive pull tension of the tendon to be repaired during arthroscopy (*R* = 0.69). In our study, elasticity measurements were associated with the arthroscopic scores, while in the Itoigawa study, elasticity measurements were associated with passive tension of the residual tendon during surgery. Although the relevant reference parameters are different, our study and their study aimed to reflect the change of stress in rotator cuff tissue after RCT through elasticity measurement.

The operation shoulders were compared to the control shoulders in our study, and the results showed that SWV-RT and SWV-RM in the operation shoulders were lower than those in the control shoulders in both the natural position and the modified crass position (*p* < 0.05). This was consistent with the results of a prospective study [30]; researchers performed an SWE examination on 80 patients with shoulder diseases, and the results showed that the SWV of the supraspinatus tendons in the tendinopathy group, partial-thickness tear group, and full-thickness tear group were 5.66 ± 0.97, 4.66 ± 1.00, and 3.78 ± 0.55 m/s, respectively, which were lower than those of the contralateral normal supraspinatus tendon (*p* < 0.05). The SWV of supraspinatus tendon tear was 4.59 ± 1.00 m/s, which was significantly lower than that of the contralateral normal supraspinatus tendon (6.68 ± 1.05 m/s).

Some researchers [13, 31, 32] believed that SWE examination could be affected by many factors, such as operator experience, instrument, patient position, and measurement site and measurement depth, so it is necessary to standardize SWE examination. Our study used the SWE measurement method recommended by a recently published review [4]. We performed a pretest before the study and demonstrated good intra- and inter-observer agreement for SWE measurements (*κ* = 0.848 and *κ* = 0.697, respectively). This result is similar to those obtained in some studies [33–35], suggesting that SWE is less operator-dependent and has good intra- and inter-observer repeatability when applied to rotator cuff tissue. In our study, the shear wave elasticity operating methods and inspection conditions were unified to minimize or avoid the measurement bias of SWE.

This study has several limitations. Firstly, it was conducted at a single center, which introduces potential subjectivity in assessing the quality score of the tendon during surgery and the repairability of the rotator cuff since all shoulder arthroscopic procedures were performed by a surgeon. Secondly, the enrolled patients had a short disease duration, and only a few patients had poor residual tendon quality (arthroscopic scores > 7). Consequently, future studies should consider expanding the sample size by including a larger number of relevant patients for multicenter investigations. This will enable further analysis of the evolving trend of SWE with disease progression and exploration of the impact of the threshold for residual tendon SWV on surgical outcomes among patients with high arthroscopic scores. Finally, only preoperative and intraoperative data were collected and analyzed in this study, so it is not possible to judge the prediction of SWE measurement on postoperative shoulder pain and retear, which needs to be further studied.

## Conclusion

Our study showed that SWE-derived tissue stiffness was negatively correlated with the quality of the residual tendon. SWV of supraspinatus tendon and muscle was a useful parameter to predict the quality of the residual tendon. These indicated that SWE could be a valuable method for preoperative evaluation of RCT residual tendon quality.

## Data Availability

All of our study data came from our institutional database. Because our institution is a military hospital, some of the patient personal information included in the study is confidential information, so we cannot provide hyperlinks to the publicly available archival dataset and original ultrasound images containing patient information. If requested, we can provide EXCEL worksheets for statistical analysis of the article data as supplementary materials.

## Abbreviations

AD: Anterior-deep site of the supraspinatus muscle
AS: Anterior-superficial site of the supraspinatus muscle
PD: Posterior-deep site of the supraspinatus muscle
PS: Posterior-superficial site of the supraspinatus muscle
RCT: Rotator cuff tear
ROI: Region of interest
SWE: Ultrasonic shear wave elastography
SWV: Shear wave velocity
SWV-RM: The SWV ratio of supraspinatus muscle
SWV-RT: The SWV ratio of supraspinatus tendon
TDD: Distal-deep site of the supraspinatus tendon
TDS: Distal-superficial site of the supraspinatus tendon
TPD: Proximal-deep site of the supraspinatus tendon
TPS: Proximal-superficial site of the supraspinatus tendon

## References

[1] Chiu YH, Chang KV, Chen IJ, Wu WT, Özçakar L (2020). Utility of sonoelastography for the evaluation of rotator cuff tendon and pertinent disorders: a systematic review and meta-analysis. Eur Radiol.

[2] Chen X, Wang Y, Chen J (2023). Clinical value of three-dimensional ultrasonography in the morphologic evaluation of rotator cuff tear: a prospective study. Eur Radiol.

[3] Bureau NJ, Deslauriers M, Lepage-Saucier M (2018). Rotator cuff tear morphologic parameters at magnetic resonance imaging: relationship with muscle atrophy and fatty infiltration and patient-reported function and health-related quality of life. J Comput Assist Tomogr.

[4] Kijowski R, Thurlow P, Blankenbaker D (2019). Preoperative MRI shoulder findings associated with clinical outcome 1 year after rotator cuff repair. Radiology.

[5] Simmer Filho J, Voss A, Pauzenberger L (2019). Footprint coverage comparison between knotted and knotless techniques in a single-row rotator cuff repair: biomechanical analysis. BMC Musculoskelet Disord.

[6] Giambini H, Hatta T, Rezaei A, An KN (2018). Extensibility of the supraspinatus muscle can be predicted by combining shear wave elastography and magnetic resonance imaging-measured quantitative metrics of stiffness and volumetric fat infiltration: a cadaveric study. Clin Biomech (Bristol, Avon).

[7] Itoigawa Y, Wada T, Kawasaki T, Morikawa D, Maruyama Y, Kaneko K (2020). Supraspinatus muscle and tendon stiffness changes after arthroscopic rotator cuff repair: a shear wave elastography assessment. J Orthop Res.

[8] Ryu KJ, Kim BH, Lee Y, Lee YS, Kim JH (2015). Modified suture-bridge technique to prevent a marginal dog-ear deformity improves structural integrity after rotator cuff repair. Am J Sports Med.

[9] Guo S, Zhu Y, Song G, Jiang C (2020). Assessment of tendon retraction in large to massive rotator cuff tears: a modified Patte classification based on 2 coronal sections on preoperative magnetic resonance imaging with higher specificity on predicting reparability. Arthroscopy.

[10] Itoigawa Y, Maruyama Y, Kawasaki T (2018). Shear wave elastography can predict passive stiffness of supraspinatus musculotendinous unit during arthroscopic rotator cuff repair for presurgical planning. Arthroscopy.

[11] Sigrist RMS, Liau J, Kaffas AE, Chammas MC, Willmann JK (2017). Ultrasound elastography: review of techniques and clinical applications. Theranostics.

[12] Cipriano KJ, Wickstrom J, Glicksman M (2022). A scoping review of methods used in musculoskeletal soft tissue and nerve shear wave elastography studies. Clin Neurophysiol.

[13] Ruby L, Mutschler T, Martini K (2019). Which confounders have the largest impact in shear wave elastography of muscle and how can they be minimized? An elasticity phantom, ex vivo porcine muscle and volunteer study using a commercially available system. Ultrasound Med Biol.

[14] Sadeghi S, Quinlan K, Eilertson KE (2019). Changes in shear modulus of the lumbar multifidus muscle during different body positions. J Biomech Eng.

[15] Lawrence RL, Ruder MC, Moutzouros V (2021). Ultrasound shear wave elastography and its association with rotator cuff tear characteristics. JSES Int.

[16] Hou SW, Merkle AN, Babb JS, McCabe R, Gyftopoulos S, Adler RS (2017). Shear wave ultrasound elastographic evaluation of the rotator cuff tendon. J Ultrasound Med.

[17] Krepkin K, Bruno M, Raya JG, Adler RS, Gyftopoulos S (2017). Quantitative assessment of the supraspinatus tendon on MRI using T2/T2* mapping and shear-wave ultrasound elastography: a pilot study. Skeletal Radiol.

[18] Martinoli C (2010). Musculoskeletal ultrasound: technical guidelines. Insights Imaging.

[19] Cofield RH, Parvizi J, Hoffmeyer PJ, Lanzer WL, Ilstrup DM, Rowland CM (2001). Surgical repair of chronic rotator cuff tears. A prospective long-term study. J Bone Joint Surg Am.

[20] Park SG, Shim BJ, Seok HG (2019). How much will high tension adversely affect rotator cuff repair integrity?. Arthroscopy.

[21] Nocera NL, Burke CJ, Gyftopoulos S, Adler RS (2021). Ultrasound-MRI correlation for healing of rotator cuff repairs using power doppler, sonographic shear wave elastography and MR signal characteristics: a pilot study. J Ultrasound Med.

[22] Yuri T, Mura N, Yuki I, Fujii H, Kiyoshige Y (2018). Contractile property measurement of the torn supraspinatus muscle using real-time tissue elastography. J Shoulder Elbow Surg.

[23] Kim SC, Shim SB, Kim WJ, Yoo JC (2022). Preoperative rotator cuff tendon integrity, tear size, and muscle atrophy and fatty infiltration are associated with structural outcomes of arthroscopic revision rotator cuff repair. Knee Surg Sports Traumatol Arthrosc.

[24] Hatta T, Giambini H, Itoigawa Y (2017). Quantifying extensibility of rotator cuff muscle with tendon rupture using shear wave elastography: a cadaveric study. J Biomech.

[25] Hatta T, Giambini H, Uehara K (2015). Quantitative assessment of rotator cuff muscle elasticity: reliability and feasibility of shear wave elastography. J Biomech.

[26] Deng H, Mi Y, Lu B, Xu P (2021). Application of virtual touch tissue imaging quantification in diagnosis of supraspinatus tendon injury. J Xray Sci Technol.

[27] Tokish JM, Hawkins RJ (2021). Current concepts in the evolution of arthroscopic rotator cuff repair. JSES Rev Rep Tech.

[28] Brage K, Juul-Kristensen B, Hjarbaek J, Boyle E, Kjaer P, Ingwersen KG (2020). Strain elastography and tendon response to an exercise program in patients with supraspinatus tendinopathy: an exploratory study. Orthop J Sports Med.

[29] Muraki T, Ishikawa H, Morise S (2015). Ultrasound elastography-based assessment of the elasticity of the supraspinatus muscle and tendon during muscle contraction. J Shoulder Elbow Surg.

[30] Kim SY, Bleakney RR, Rindlisbacher T, Ravichandiran K, Rosser BWC, Boynton E (2013). Musculotendinous architecture of pathological supraspinatus: a pilot in vivo ultrasonography study. Clin Anat.

[31] Brandenburg JE, Eby SF, Song P (2014). Ultrasound elastography: the new frontier in direct measurement of muscle stiffness. Arch Phys Med Rehabil.

[32] Seo JB, Yoo JS, Ryu JW (2014). The accuracy of sonoelastography in fatty degeneration of the supraspinatus: a comparison of magnetic resonance imaging and conventional ultrasonography. J Ultrasound.

[33] Ewertsen C, Carlsen JF, Christiansen IR, Jensen JA, Nielsen MB (2016). Evaluation of healthy muscle tissue by strain and shear wave elastography - dependency on depth and ROI position in relation to underlying bone. Ultrasonics.

[34] Klauser AS, Miyamoto H, Bellmann-Weiler R, Feuchtner GM, Wick MC, Jaschke WR (2014). Sonoelastography: musculoskeletal applications. Radiology.

[35] Hackett L, Aveledo R, Lam PH, Murrell GA (2020). Reliability of shear wave elastography ultrasound to assess the supraspinatus tendon: an intra and inter-rater in vivo study. Shoulder Elbow.

